# Supplementary material for: Ulceroprotective Effects of Epilobium angustifolium Extract in DSS-Induced Colitis in Mice
Source: Curr Issues Mol Biol. 2025 Jun 10;47(6):444. doi: 10.3390/cimb47060444 (PMC12191838; doi:10.3390/cimb47060444)
Supplement: Supplementary file 1 [file cimb-47-00444-s001.zip › cimb-3673946-supplementary.pdf]

Ulceroprotective Effects of *Epilobium angustifolium* Extract in DSS-Induced Colitis in Mice

Rumyana Simeonova [1,*], Rositsa Mihaylova [1], Reneta Gevrenova [2], Ionko Savov [3] and Dimitrina Zheleva-Dimitrova [2,*]

[1] Department of Pharmacology, Pharmacotherapy and Toxicology, Faculty of Pharmacy, Medical University of Sofia, 1000 Sofia, Bulgaria; rmihaylova@pharmfac.mu-sofia.bg
[2] Department of Pharmacognosy, Faculty of Pharmacy, Medical University of Sofia, 1000 Sofia, Bulgaria; rgevrenova@pharmfac.mu-sofia.bg
[3] Institute of Emergency Medicine "N. I. Pirogov", Bul. Totleben 21, 1000 Sofia, Bulgaria; yonko_savov@hotmail.com
* Correspondence: rsimeonova@pharmfac.mu-sofia.bg (R.S.); dzheleva@pharmfac.mu-sofia.bg (D.Z.-D.)

Supplemental Materials

Table S1. Secondary metabolites in *Epilobium angustifolium* methanol-aqueous extracts.

No	Identified/Tentatively Annotated Compound	Molecular Formula	Exact Mass [M-H]$^-$	t_R (min)	Δ ppm
**Hydroxybenzoic, Hydroxycinnamic Acids, Phenylethanoid Glycosides, and Coumarins**					
1.	galloyl *O*-hexose	$C_{13}H_{16}O_{10}$	331.0671	0.94	1.511
2.	gallic acid [a]	$C_7H_6O_5$	169.0142	1.14	6.133
3.	gallic acid *O*-hexoside 1	$C_{13}H_{16}O_{10}$	331.0687	1.17	1.511
4.	hydroxybenzoic acid-*O*-hexoside	$C_{13}H_{16}O_8$	299.0778	1.26	−2.543
5.	gallic acid *O*-hexoside 2	$C_{13}H_{16}O_{10}$	331.0687	1.56	1.118
6.	protocatechuic acid-*O*-hexoside	$C_{13}H_{16}O_9$	315.0727	1.68	1.221
7.	vanillic acid-*O*-hexoside	$C_{14}H_{18}O_9$	329.0875	1.77	7.581
8.	protocatechuic acid [a]	$C_7H_6O_4$	153.0181	2.02	−1.392
9.	*p*-hydroxyphenylacetic acid *O*-hexoside	$C_{14}H_{18}O_8$	313.0932	2.13	0.988
10.	hydroxybenzoyl hexose	$C_{13}H_{16}O_8$	299.0778	2.16	0.600
11.	syringic acid-*O*-hexoside	$C_{15}H_{20}O_{10}$	359.0985	2.26	2.311
12.	caffeic acid-*O*-hexoside 1	$C_{15}H_{18}O_9$	341.0871	2.40	−2.332
13.	caffeic acid-*O*-hexoside 2	$C_{15}H_{18}O_9$	341.0871	2.61	−3.827
14.	caffeoyl-*O*-hexose	$C_{15}H_{18}O_9$	341.0871	2.82	−5.469
15.	4-hydroxybenzoic acid [a]	$C_7H_6O_3$	137.0230	2.84	−10.052
16.	gentisic acid *O*-hexoside	$C_{13}H_{16}O_9$	315.0727	2.84	1.221
17.	*p*-coumaric acid [a]	$C_9H_8O_3$	163.0389	3.01	−6.792
18.	caffeic acid *O*-hexoside 3	$C_{15}H_{18}O_9$	341.0871	3.07	−0.602
19.	methylgallate	$C_8H_8O_5$	183.0299	3.15	0.711
20.	quinic acid	$C_7H_{12}O_6$	191.0549	3.19	−5.817
21.	umbeliferone	$C_9H_6O_3$	161.0244	3.19	−7.250
22.	coumaric acid-*O*-hexoside	$C_{15}H_{18}O_8$	325.0930	3.34	−3.355
23.	caffeic acid [a]	$C_9H_8O_4$	179.0339	3.54	−6.044
24.	*o*-coumaric acid [a]	$C_9H_8O_3$	163.0389	4.55	−7.467

25.	galloyl-(caffeoyl)-hexose	$C_{22}H_{22}O_{13}$	493.0988	4.78	3.643
26.	salicylic acid [a]	$C_7H_6O_3$	137.0230	6.29	−10.125
	**Mono- and Diacylquinic Acids**				
27.	3-galloylquinic acid	$C_{14}H_{16}O_{10}$	343.0671	1.25	1.370
28.	neochlorogenic (3-caffeoylquinic) acid [a]	$C_{16}H_{18}O_9$	353.0867	2.36	1.458
29.	3-*p*-coumaroylquinic acid	$C_{16}H_{18}O_8$	337.0928	3.01	1.096
30.	chlorogenic (5-caffeoylquinic) acid [a]	$C_{16}H_{18}O_9$	353.0874	3.19	0.665
31.	4-caffeoylquinic acid	$C_{16}H_{18}O_9$	353.0878	3.37	−0.100
32.	3-feruloylquinic acid	$C_{17}H_{20}O_9$	367.1034	3.44	1.157
33.	1-galloyl-3-caffeoylquinic acid	$C_{23}H_{22}O_{13}$	505.09	3.58	2.348
34.	4-*p*-coumaroylquinic acid	$C_{16}H_{18}O_8$	337.0928	3.79	
35.	5-caffeoylquinic acid isomer	$C_{16}H_{18}O_9$	353.0874	3.88	1.684
36.	5-*p*-coumaroylquinic acid	$C_{16}H_{18}O_8$	337.0928	3.95	1.629
37.	1-caffeoyl-3-galloylquinic acid	$C_{23}H_{22}O_{13}$	505.0988	4.10	1.913
38.	1-galloyl-5-caffeoylquinic acid	$C_{23}H_{22}O_{13}$	505.0988	4.19	2.032
39.	5-feruloylquinic acid	$C_{17}H_{20}O_9$	367.1034	4.39	1.402
40.	3-caffeoyl-5-galloylquinic acid	$C_{23}H_{22}O_{13}$	505.0988	4.56	1.735
41.	5-*p*-coumaroylquinic acid isomer	$C_{16}H_{18}O_8$	337.0928	4.61	1.926
42.	caffeoyl-hydroxybenzoylquinic acid	$C_{23}H_{24}O_{12}$	491.1196	4.89	1.691
43.	5-feruloylquinic acid isomer	$C_{17}H_{20}O_9$	367.1034	4.91	0.939
44.	3-galloyl-5-*p*-coumaroylquinic acid	$C_{23}H_{22}O_{12}$	489.1038	5.48	1.555
45.	3-feruloyl-5-galloylquinic acid	$C_{26}H_{32}O_{11}$	519.1872	6.70	0.954
	**Tannins**				
46.	galloyl-HHDP-hexose 1	$C_{27}H_{22}O_{18}$	633.0733	1.11	2.074
47.	galloyl-HHDP-hexose 2	$C_{27}H_{22}O_{18}$	633.0733	1.40	2.264
48.	digalloyl-hexose 1	$C_{20}H_{20}O_{14}$	483.0780	1.60	1.266
49.	digalloyl-hexose 2	$C_{20}H_{20}O_{14}$	483.0780	2.60	1.204
50.	oenothein B 1	$C_{68}H_{46}O_{44}$	1568.1518	2.74	1.743
51.	digalloyl-HHDP-hexose (tellimagrandin I) 1	$C_{34}H_{26}O_{22}$	785.0843	3.01	2.630
52.	digalloyl-hexose 3	$C_{20}H_{20}O_{14}$	483.0780	3.09	1.659
53.	galloyl-HHDP- hexose 3	$C_{27}H_{22}O_{18}$	633.0733	3.24	2.169
54.	oenothein B 2	$C_{68}H_{46}O_{44}$	1568.1518	3.33	1.590
55.	brevifolin carboxylic acid	$C_{13}H_8O_8$	291.0149	3.34	1.511
56.	digalloyl-hexose 4	$C_{20}H_{20}O_{14}$	483.0780	3.35	1.390
57.	oenothein A1	$C_{102}H_{70}O_{66}$	2352.2277	3.44	2.250
58.	digalloyl-HHDP-hexose (tellimagrandin I) 2	$C_{34}H_{26}O_{22}$	785.0843	3.66	1.776
59.	oenothein A2	$C_{102}H_{70}O_{66}$	2352.2277	3.89	1.731
60.	trigalloyl-hexose 1	$C_{27}H_{24}O_{18}$	635.0890	3.92	2.209
61.	trigalloyl-hexose 2	$C_{27}H_{24}O_{18}$	635.0890	4.01	2.304
62.	trigalloyl-hexose 3	$C_{27}H_{24}O_{18}$	635.0890	4.11	1.926
63.	trigalloyl-hexose 4	$C_{27}H_{24}O_{18}$	635.0890	4.31	2.304
64.	tellimagradin II 1	$C_{41}H_{30}O_{26}$	937.0953	4.57	1.796
65.	ellagic acid *O*-pentoside	$C_{19}H_{14}O_{12}$	433.0412	4.66	0.811
66.	tellimagradin II 2	$C_{41}H_{30}O_{26}$	937.0953	4.74	1.726
67.	tetragalloyl-hexose 1	$C_{34}H_{28}O_{22}$	787.0999	4.94	1.518
68.	ellagic acid[a]	$C_{14}H_6O_8$	300.9991	5.01	−0.101

69.	tetragalloyl-hexose 2	$C_{34}H_{28}O_{22}$	787.0999	5.05	2.521
	Flavonoids				
70.	procyanidin dimer	$C_{30}H_{26}O_{12}$	577.1351	2.92	2.617
71.	catechin/epicatechin	$C_{15}H_{14}O_{6}$	289.0718	3.12	0.583
72.	myricetin 3-*O*-galloylhexoside	$C_{28}H_{24}O_{17}$	631.0941	4.19	1.914
73.	kaempferol 7-O-deoxyhexosylhexoside 1	$C_{27}H_{30}O_{15}$	593.1512	4.27	2.254
74.	patuletin 3-*O*-dihexoside	$C_{28}H_{32}O_{18}$	655.1516	4.37	1.989
75.	myricetin 3-*O*-hexoside1	$C_{21}H_{20}O_{13}$	479.0831	4.48	−0.217
76.	myricetin *O*-hexuronide	$C_{21}H_{18}O_{14}$	493.0624	4.51	1.423
77.	myricetin 3-*O*-hexoside 2	$C_{21}H_{20}O_{13}$	479.0831	4.57	0.994
78.	6-hydroxykaempferol methyl ether *O*-dihexoside	$C_{28}H_{32}O_{17}$	639.1567	4.77	2.233
79.	quercetin 3-*O*-galloylhexoside 1	$C_{28}H_{24}O_{16}$	615.0992	4.82	1.727
80.	kaempferol *O*-dihexoside	$C_{27}H_{30}O_{16}$	609.1461	4.85	2.023
81.	quercetin galloylhexoside 2	$C_{28}H_{24}O_{16}$	615.0992	4.96	1.825
82.	myricetin 3-*O*-deoxyhexoside	$C_{21}H_{20}O_{12}$	463.0882	5.11	1.384
83.	6-hydroxykaempferol methyl ether O-deoxyhexosylhexoside	$C_{28}H_{32}O_{16}$	623.1618	5.14	1.929
84.	Isoquercitrin [a]	$C_{21}H_{20}O_{12}$	463.0885	5.19	1.319
85.	quercetin *O*-hexuronide	$C_{21}H_{18}O_{13}$	477.0675	5.22	1.543
86.	hyperoside [a]	$C_{21}H_{20}O_{12}$	463.0885	5.29	1.837
87.	kaempferol-galloylhexoside 1	$C_{28}H_{24}O_{15}$	599.1042	5.29	2.148
88.	quercetin *O*-pentoside	$C_{20}H_{18}O_{11}$	433.0776	5.62	0.982
89.	kaempferol 3-*O*-glucoside [a]	$C_{21}H_{20}O_{11}$	447.0934	5.65	2.607
90.	kaempferol-galloylhexoside 2	$C_{28}H_{24}O_{15}$	599.1042	5.67	2.148
91.	myricetin 3-*O*-caffeoylhexoside 1	$C_{22}H_{22}O_{12}$	641.1160	5.71	1.813
92.	kaempferol *O*-hexuronide	$C_{21}H_{18}O_{12}$	461.0725	5.83	1.368
93.	myricetin 3-*O*-caffeoylhexoside2	$C_{22}H_{22}O_{12}$	641.1160	5.85	1.906
94.	quercetin 3-*O*-deoxyhexoside	$C_{21}H_{20}O_{11}$	447.0933	5.90	1.645
95.	isorhamnetin 3-*O*-glucoside [a]	$C_{22}H_{22}O_{12}$	477.1038	6.02	2.831
96.	naringenin *O*-hexoside	$C_{21}H_{22}O_{10}$	433.1140	6.06	5.703
97.	isorhamnetin O-hexuronide	$C_{22}H_{20}O_{13}$	491.0831	6.08	1.397
98.	kaempferol 3-*O*-pentoside	$C_{20}H_{18}O_{10}$	417.0827	6.08	0.530
99.	kaempferol *O*-deoxyhexosylhexoside 2	$C_{27}H_{30}O_{15}$	593.1512	6.32	1.697
100.	myricetin *O*-*p*-coumaroylhexoside	$C_{30}H_{26}O_{15}$	625.1199	6.59	1.819
101.	kaempferol *O*-deoxyhexoside	$C_{21}H_{20}O_{10}$	431.0984	6.59	0.380
102.	myricetin 3-O-feruloylhexoside	$C_{31}H_{28}O_{16}$	655.1305	6.74	2.659
103.	kaempferol 7-*O*-caffeoylhexoside	$C_{30}H_{26}O_{14}$	609.1250	6.81	1.940
104.	quercetin *O*-coumaroylhexoside 1	$C_{30}H_{26}O_{14}$	609.1250	7.05	0.643
105.	quercetin *O*-coumaroylhexoside 2	$C_{30}H_{26}O_{14}$	609.1250	7.17	1.743
106.	quercetin 3-*O*-feruloylhexoside 1	$C_{31}H_{28}O_{15}$	639.1355	7.20	1.841
107.	quercetin 3-*O*-feruloylhexoside 2	$C_{31}H_{28}O_{15}$	639.1355	7.32	1.841
108.	quercetin *O*-coumaroylhexoside 3	$C_{30}H_{26}O_{14}$	609.1250	7.54	1.743
109.	kaempferol *O*-coumaroylhexoside 1	$C_{30}H_{26}O_{13}$	593.1301	7.58	2.168
110.	quercetin [a]	$C_{15}H_{10}O_{7}$	301.0354	7.62	
111.	kaempferol *O*-coumaroylhexoside 2	$C_{30}H_{26}O_{13}$	593.1301	7.68	2.084
112.	kaempferol O-feruloylhexoside 1	$C_{31}H_{28}O_{14}$	623.1406	7.71	2.345

113.	kaempferol O-feruloylhexoside 2	$C_{31}H_{28}O_{14}$	623.1406	7.82	
114.	6-hydroxykaempferol methyl ether	$C_{16}H_{12}O_7$	315.0510	8.82	1.441
115.	naringenin [a]	$C_{15}H_{12}O_5$	271.0612	8.58	0.468
116.	kaempferol [a]	$C_{15}H_9O_7$	285.0406	8.83	−0.215
117.	quercetin *O*-cinnamoylhexoside 1	$C_{30}H_{26}O_{13}$	593.1301	8.85	2.067
118.	quercetin *O*-cinnamoylhexoside 2	$C_{30}H_{26}O_{13}$	593.1301	9.12	2.084
119.	isorhamnetin [a]	$C_{16}H_{12}O_7$	315.0510	9.10	1.441
120.	kaempferol *O*-cinnamoylhexoside	$C_{30}H_{26}O_{12}$	577.1351	9.50	−0.310
121.	myricetin [a]	$C_{15}H_{10}O_8$	In (+)ESI-MS/MS 319.0448	9.49	−0.804

a—Identified by comparison with an authentic standard.

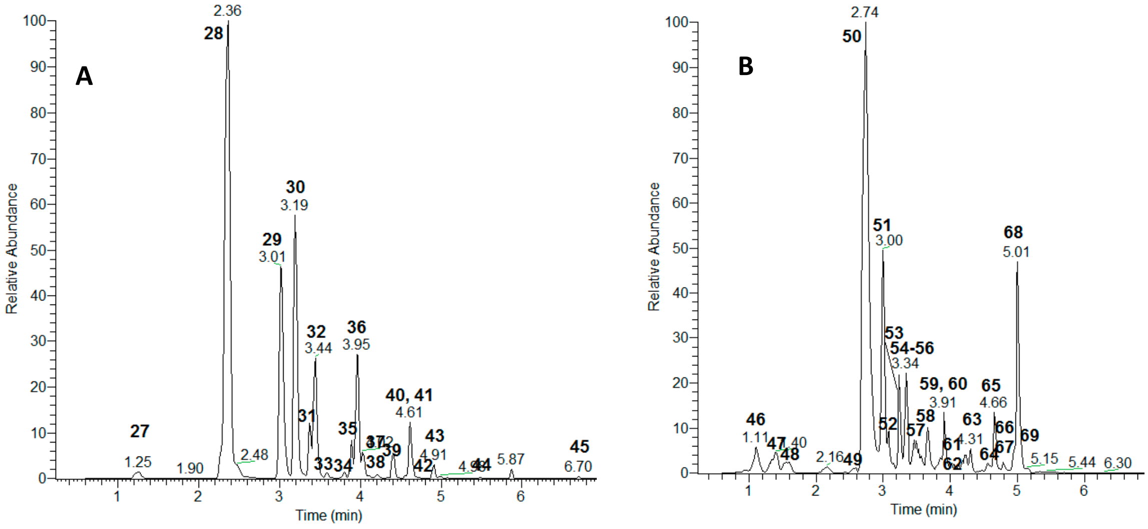

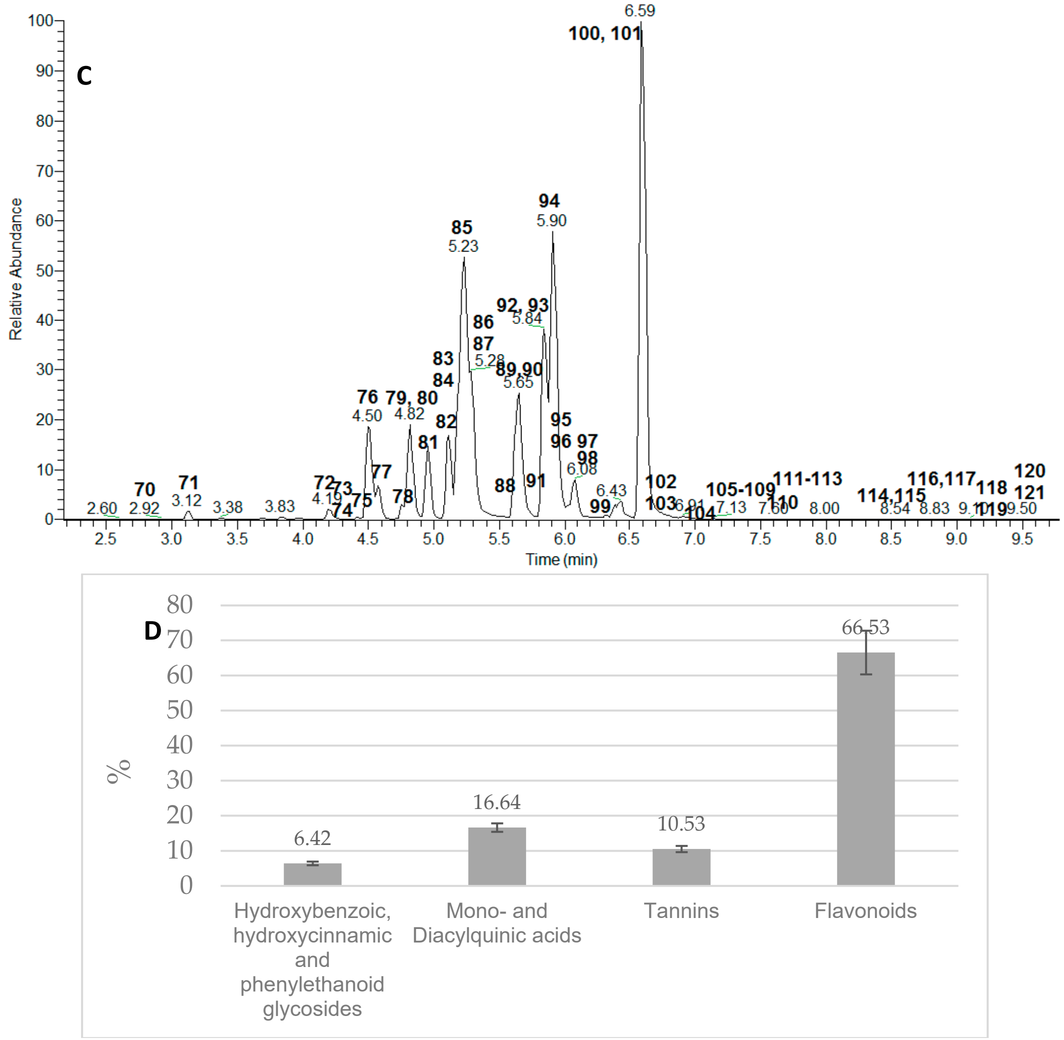

Figure S1. Extracted ion chromatograms of acylquinic acids (**A**), tannins (**B**), and flavonoids (**C**) in the *Epilobium angustifolium* extract; the percentage ratio of the main classes' secondary metabolites (**D**). For peak numbering, see Table S1.
